# *Salix* transect of Europe: structured genetic variation and isolation-by-distance in the nettle psyllid, *Trioza urticae* (Psylloidea, Hemiptera), from Greece to Arctic Norway

**DOI:** 10.3897/BDJ.5.e10824

**Published:** 2017-01-13

**Authors:** Rungtip Wonglersak, Quentin Cronk, Diana Percy

**Affiliations:** 1 University College London, London, United Kingdom; 2 University of British Columbia, Vancouver, Canada; 3 Natural History Museum, London, United Kingdom

**Keywords:** biogeography, haplotype network, latitudinal cline, isolation by distance, megatransect, Triozidae

## Abstract

**Background:**

The common nettle (*Urtica dioica* L.) is co-associated with willows (*Salix* spp.) in riparian habitats across Europe. We sampled the widespread nettle psyllid, *Trioza urticae* (Linné, 1758), from *Urtica* in willow habitats on a megatransect of Europe from the Aegean to the Arctic Ocean. The aim of this study was to use an unusually widespread insect to assess the influence of geographic distances and natural geographic barriers on patterns of genetic variation and haplotype distribution.

**New information:**

Phylogeographic analysis using DNA sequences of two mtDNA regions, COI and cytB, shows that *T. urticae* specimens are organized into four regional groups (southern, central, northern and arctic). These groups are supported by both phylogenetic analysis (four geographically-based clades) and network analysis (four major haplotype groups). The boundary between southern and central groups corresponds to the Carpathian Mountains and the boundary between the central and northern groups corresponds to the Gulf of Finland. Overall these groups form a latitudinal cline in genetic diversity, which decreases with increasing latitude.

## Introduction

A transect of Europe that sampled *Salix* (willow) habitats from the Aegean to the Arctic ocean has already been described [Cronk et al. 2015]. The common nettle (*Urtica dioica* L.) is associated with the eutrophic riparian habitats favoured by willows, and variation in *Urtica* samples along this transect has also been described [Cronk et al. 2016]. The co-occurrence of *Salix* and *Urtica* allowed sampling of insects on both plant groups [e.g., Canty et al. 2016], including the sampling for this study of the common nettle psyllid, *Trioza urticae* (Linné, 1758) (Psylloidea, family Triozidae), which is found at nearly every site along the transect (32 out of 42 sites). Members of the hemipteran superfamily Psylloidea are small, phloem-feeding, oligophagous insects [Hodkinson 2009], generally known as psyllids, or jumping plant lice. They are organized into eight families [Burckhardt and Ouvrard 2012]. *Trioza urticae* is one of the most common European psyllid species with a wide distribution across the Palaearctic [Ouvrard 2016]. *Trioza urticae* is a host-specific herbivore that feeds on *Urtica dioica*, the common stinging nettle, and a few related species [Ouvrard 2016].

The aim of this study is to take advantage of a widely dispersed pan-European insect, together with a megatransect approach to sampling to assess population structuring across Europe, particularly in relation to latitude. We wish in particular to determine the effect of biogeographic barriers across Europe on genetic structuring of *T. urticae* populations.

## Materials and Methods

### Specimen selection

**Figure 1.**
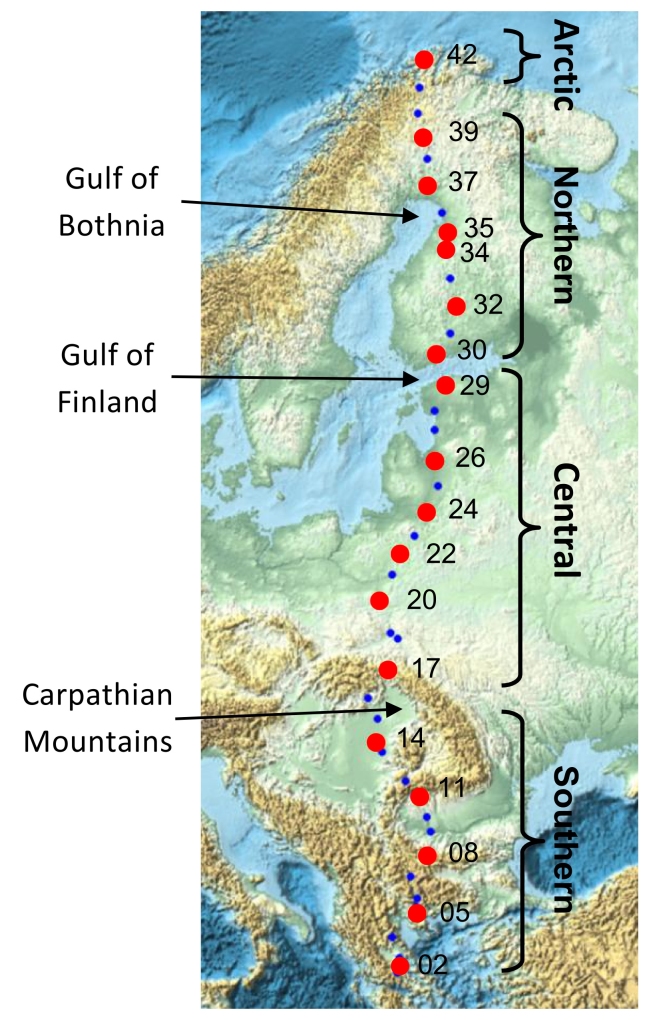
Map showing all 42 sites along an eastern European megatransect. *Trioza urticae* samples were selected from 18 sites (red points) along the transect. Three major natural geographic barriers are indicated, and the four regional partition of sites (southern, central, northern, arctic) referred to in the genetic analyses are shown.

From the 42 sites sampled along the *Salix* transect of Europe [Cronk et al. 2015], 18 sites were chosen for genetic analysis of *Trioza urticae* collected from common stinging nettle, *Urtica dioica* [Cronk et al. 2016], covering Greece, Bulgaria, Romania, Hungary, Poland, Lithuania, Latvia, Estonia, Finland and Norway (Table 1; Fig. 1). Additionally, a supplementary site from Greece was included, as well as three sites from western Europe for comparison. This investigation of variation along the transect is based on an analysis of 87 specimens sequenced from Eastern Europe with nine additional specimens from western Europe. Typically five specimens of *T. urticae* were selected from each site along the transect (Table 1). All samples were preserved in the field in 95 percent ethanol.

### Sequencing

DNAs from 96 specimens, including 87 samples from the eastern European megatransect and 9 samples from western Europe, were extracted using a Qiagen DNeasy Blood and Tissue Kit [QIAGEN Ltd., Manchester, UK]. Two regions of mtDNA; cytochrome c oxidase subunit I (COI) and cytochrome B (cytB) were amplified using standard PCR procedures as described previously [Percy 2003, Percy et al. 2015]. The amplified PCR products were then sequenced using the Sanger sequencing method [Sanger et al. 1977]. The bidirectional sequences (forward and reverse) were then assembled, edited, and trimmed in Sequencher® version 5.4.5 [Gene Codes Corporation, Ann Arbor, MI USA]. Sequences for COI (472bp) and cytB (385bp) were then imported to Se-Al software (version 2.0) [Rambaut 1996] to view and check alignments before being exported to FASTA format. These regions were concatenated using Sequence Matrix software [Vaidya et al. 2011] and exported in NEXUS format. The resulting NEXUS file was imported into PAUP* software (version 4.0) [Swofford 2002] to perform phylogenetic analyses using neighbour-joining (NJ) to determine sequence divergence, and maximum parsimony (MP). For 5 of the 87 transect samples, sequences were recovered for only one of the two gene regions. These five samples were included in the MP consensus analysis, but excluded from the maximum likelihood (ML), haplotype and regional clade analyses described below. The DNA sequences are deposited in GenBank with accession numbers KY011106-KY011201 (COI) and KY011202-KY011296 (cytB).

### Data analysis

To determine regional clade structure for the 82 transect samples, a NJ analysis (p-distance) with 1,000 bootstrap replicates was performed in PAUP*, and a maximum likelihood (ML) analysis was conducted with 100 rapid bootstrap replicates using RAxML (version 8.2.4) [Stamatakis 2014] and run on CIPRES Science Gateway [Miller et al. 2010]. In order to include all 96 samples (including complete and partially sequenced samples), a maximum parsimony analysis (MP) was performed with 1,000 random addition replicates, nearest neighbour interchange (NNI) branch swapping and a 50 percent majority-rule tree derived from the consensus of 51,397 MP trees using PAUP*. The resulting cladogram was rooted with two outgroup taxa in the genus *Bactericera* (family Triozidae): *B. cockerelli* (Šulc, 1909) and *B. albiventris* (Foerster, 1848).

To analyse haplotype variation across the latitudinal gradient, the DNA sequences were used to create a haplotype median-joining network [Bandelt et al. 1999] and a haplotype map using PopART (version 1.7) [Leigh et al. 2015]. Population statistics were then derived from an analysis of molecular variation (AMOVA) using F-statistic analogues (phi-st: φ*ST* and phi-ct: φ*CT*) [Excoffier et al. 1992] as a mtDNA measure of the proportion of nucleotide diversity among subpopulations, relative to the total. Subpopulations were determined either as sites in a simple AMOVA, or as the four regional clades in a nested AMOVA.

Latitudinal clines of genetic variation were assessed based on the concept of “isolation by distance” (IBD) [Wright 1943]. A pairwise genetic distance matrix (p-distances) was generated in PAUP*, and a pairwise geographic distance matrix was generated using the latitude and longitude coordinates (GPS point data transformed into pairwise distances using the Geographic Distance Matrix Generator; [Ersts 2012]). To test the correlation between the genetic and geographic matrices, a Mantel test [Manly 2004] (1000 iterations) was performed with the ‘Isolation-by-Distance Web Service’ (IBDWS version 3.23) [Jensen et al. 2005], and Reduced Major Axis (RMA) regression used to assess the slope and intercept of the relationship among these variables.

## Results

### Genetic variation and phylogeography

**Figure 2.**
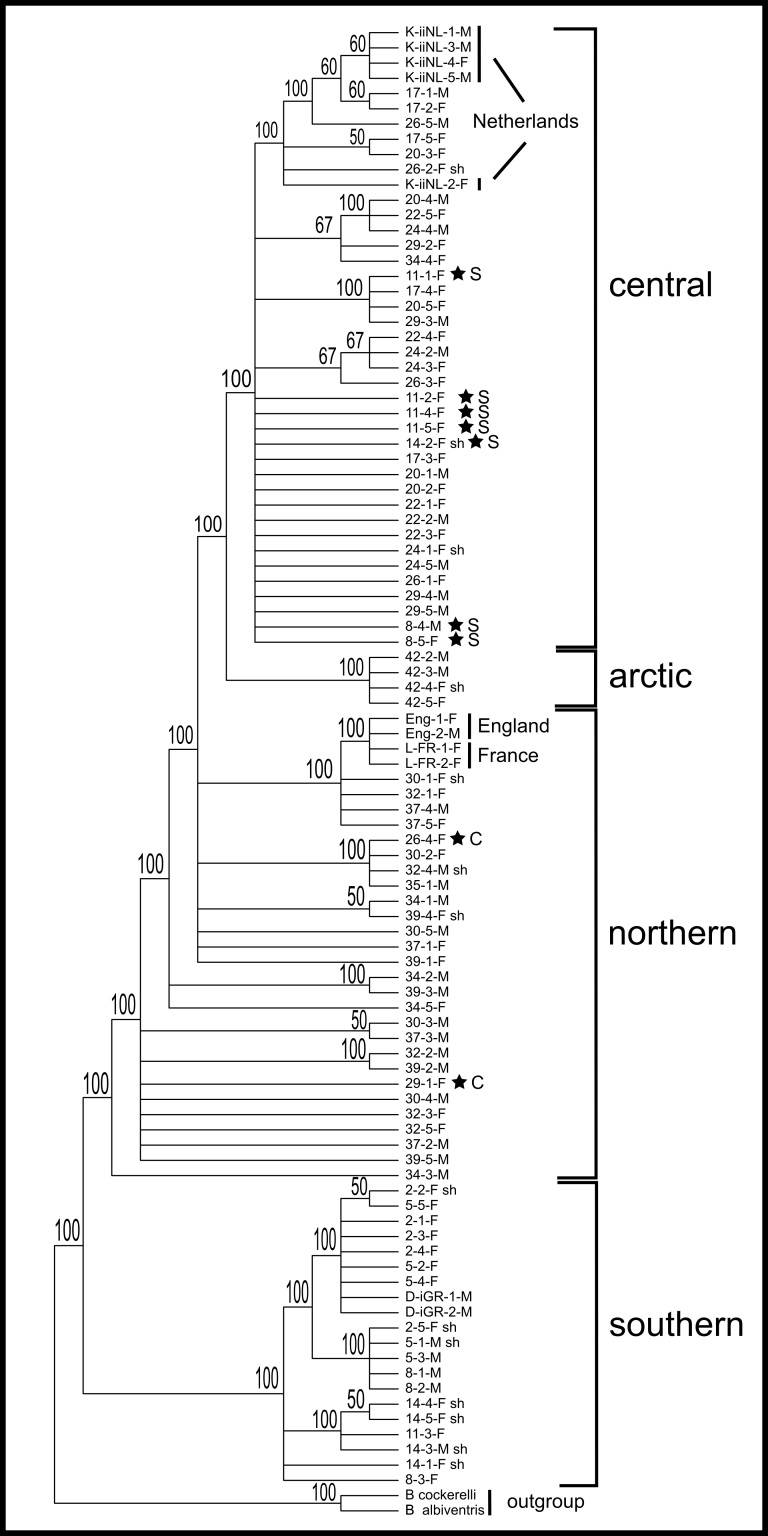
Majority-rule consensus tree of the MP analysis incorporating all 96 samples. The four regional clades as determined from the ML and haplotype analyses are indicated. Stars indicate the position of individuals within regional clades but not from that region (see also ML and haplotype network in Figs 3, 5, 6). The root is provided by two outgroup taxa in the genus *Bactericera*.

**Figure 3.**
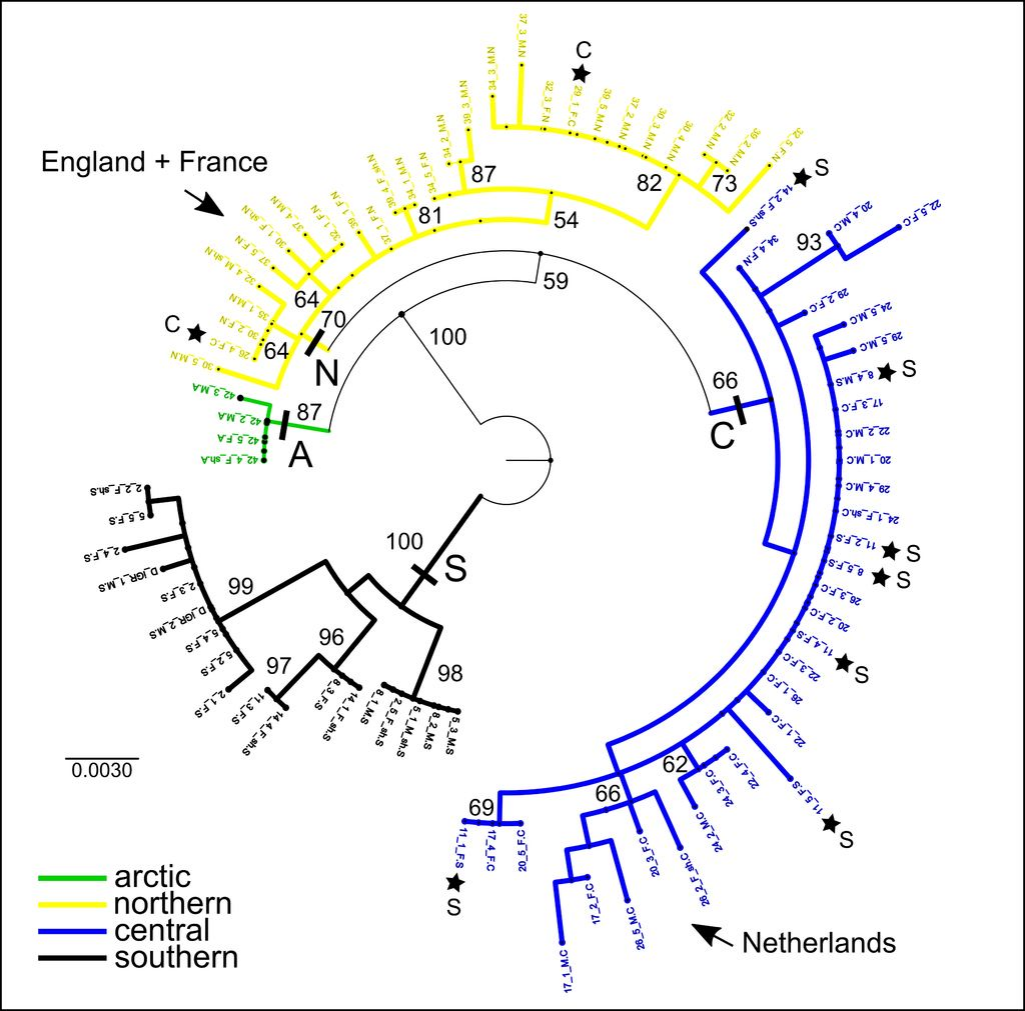
The ML tree of 82 samples along the transect showing bootstrap support values. Four regional clades are identified (southern, central, northern, arctic) and the clade position of the non-included western European samples is indicated. Stars indicate the position of individuals within regional clades but not from that region (see also MP and haplotype network in Figs 2, 5, 6).

**Figure 4.**
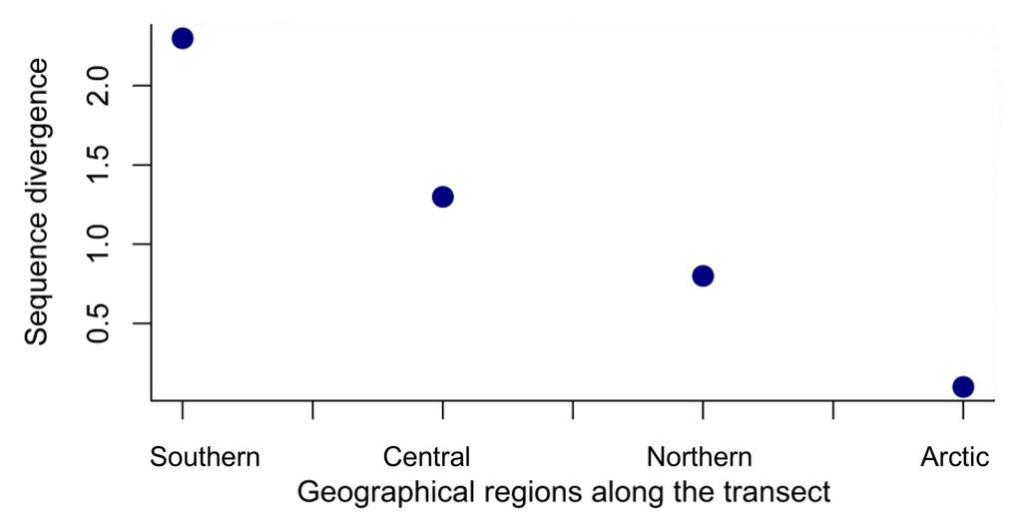
Sequence divergence (p-distances) within four major geographical regions along an eastern European megatransect.

The 857bp matrix of all 96 samples contains 37 parsimony informative characters, 36 of which are found among the transect samples, and the addition of the nine western European individuals adds only one additional informative character. Total sequence divergence (p-distances) for all 96 samples was 2.8%. Each of the phylogenetic analyses are able to separate populations into regional groups. The consensus analysis using MP with all 96 samples recovers three of the four regional groups (Fig. 1, Fig. 2), and NJ and ML analyses (best ML tree score -lnL = -3031.39) using 82 transect samples (with complete sequences for both gene regions) separate populations into four regional groups (Fig. 1, Fig. 3). There is a progressive decline in sequence divergence northwards along the transect, and sequence divergence within the major geographic regions of the transect was: southern 2.3%, central 1.3%, northern 0.8%, arctic 0.1% (Fig. 4).

In total, 48 haplotypes were found using 82 samples along the transect. The sites with the most sequence divergence among individuals (>1.5%) are all southern sites: site 11 (1.9%), site 14 (1.8%), and site 8 (1.6%). This is due to some samples from these sites also clustering within the central region clade. Among the other regions the most diverse site is a central region site, 26 (1.1%), which has some individuals clustering in the northern region clade (Figs 2, 3). Interestingly, the samples included from western Europe do not cluster together, instead the Netherlands samples cluster with the regionally central clade and the samples from England and France cluster with the regionally northern clade (Figs 2, 3).

### Analyses of molecular variation between and within sites

The simple AMOVA found 70.4 percent variation between sites, and 29.6 percent variation within sites. The nested AMOVA found 61.3 percent variation between regional groups, 14.9 percent variation between sites within groups, and 23.81 percent within sites within groups. The φ*ST*, which is a measure of variance among populations relative to total variance, shows significant differentiation among the populations across the transect (φ*ST* = 0.76, p < 0.001, 1000 permutations). This confirms that there is substantial population structure in the data even though the overall mtDNA sequence divergence is less than 3 percent. Furthermore, φ*CT*, which is a measure of variance among regional groups relative to total variance, also shows highly significant differentiation among the regional groups of the transect (φ*CT* = 0.61, p < 0.001, 1000 permutations).

### Haplotype diversity

**Figure 5.**
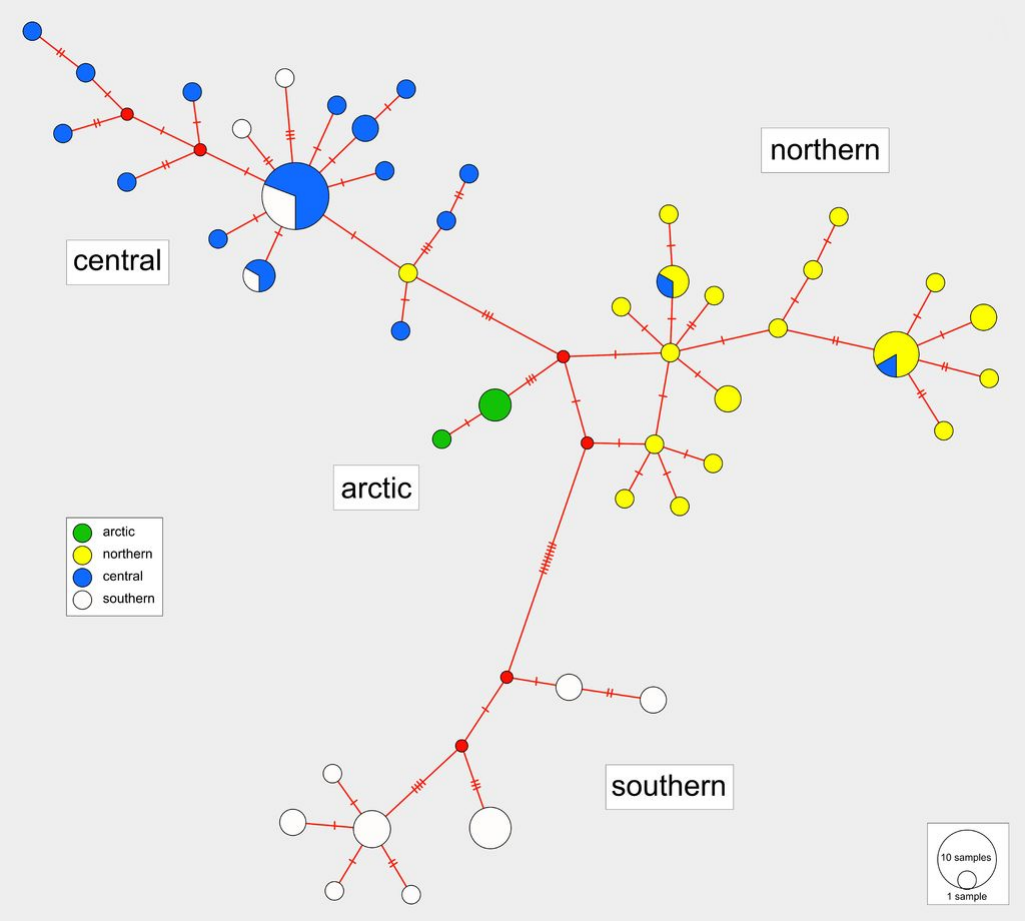
Median-joining haplotype network with colours indicating haplotype distributions by region (southern, central, northern, arctic), with region allocation determined from a ML clade analysis of the DNA sequence data.

**Figure 6.**
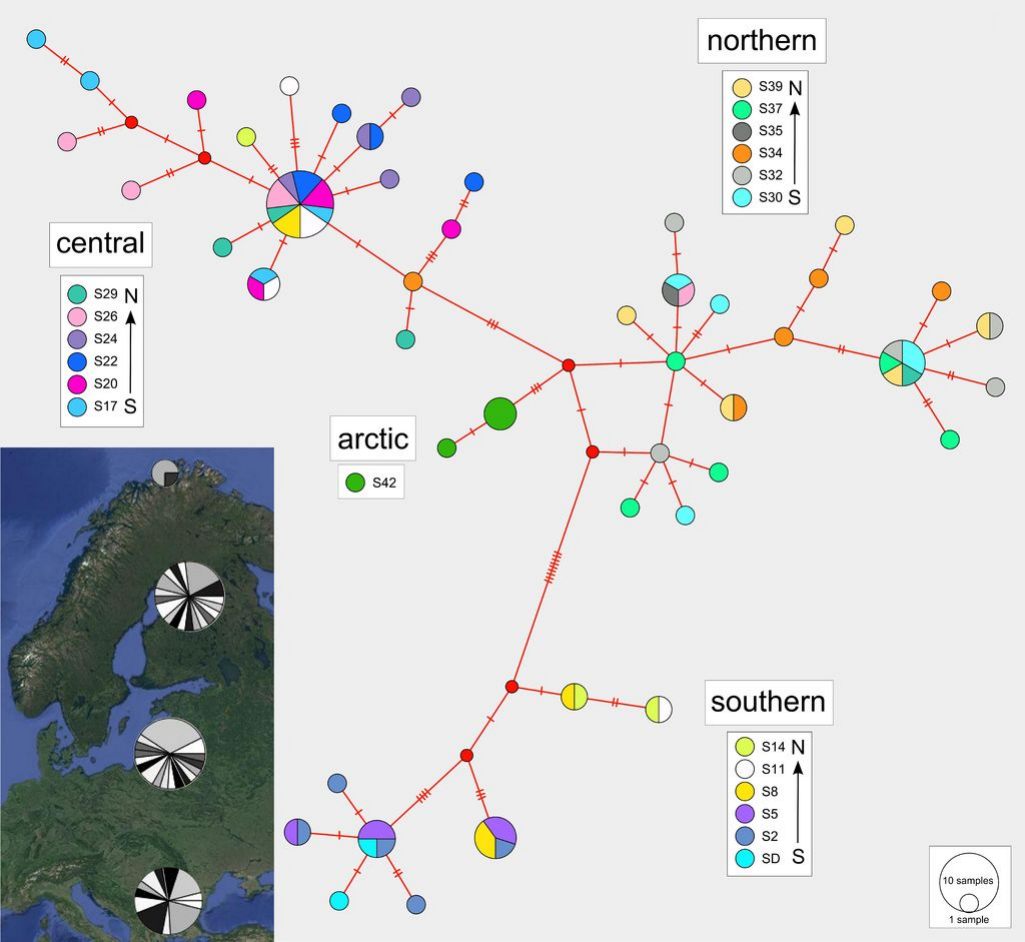
Median-joining haplotype network with colours indicating haplotype distributions by site. Labelled regions (southern, central, northern, arctic) are determined from a ML clade analysis of the DNA sequence data. Inset: Indication of the proportions of haplotypes represented within each of the four major regions.

The haplotype median-joining network reflects the four regional clusters found in the phylogenetic analyses (Figs 5, 6). There are a higher number of unique haplotypes found within the central and northern regions (Fig. 6), but the southern group is the most divergent from the other groups (Figs 5, 6). Haplotype variation in the arctic region is very low, but this region also includes fewer samples.

### Isolation by distance

A Mantel test of the correlation between genetic distance (calculated from the DNA sequences) and geographic distance data (calculated from the latitude and longitude coordinates) exhibited high significant positive results (r = 0.5295, p < 0.001). This indicates that genetic similarity declines with increasing geographic distance.

## Discussion

### Genetic variation

Four regional groups were discovered using genetic analysis and a latitudinal cline of the genetic diversity of these groups is evident with lower genetic diversity at high latitudes. The arctic region has lowest genetic diversity consistent with it being a marginal area of occurrence for the species. Variation of genetic diversity with latitude has been pointed out before [Eckert et al. 2008; Sotka et al. 2004; Adams and Hadly 2012] and this appears to be a general pattern, confirmed here. Possible explanations include a migrational filter on variation during post-glacial recolonization from south to north [Hewitt 2004]. Another factor is that warmer climates may have higher rates of mutation, and multiple and/or shorter generation times [Rohde 1992], possibly resulting in higher intraspecific genetic divergence.

### Genetic structure

High value of φ*CT* indicates that four regional groups across the transect are highly structured with limited gene flow occurring among these groups. Although adult psyllids are winged allowing active migration, biogeographical barriers along the transect possibly restrict gene flow among these four groups. The barrier between southern and central groups (between sites 14 to 17) is coincident with the Carpathian Mountains, a major biogeographic boundary in Europe. The boundary between the central and northern groups (sites 29 to 30) is coincident with the Gulf of Finland, and approximately to the temperate to boreal transition in Europe. Consistent with the general assumption that genetic differentiation increases with increasing geographic distance [Wright 1943; Rousset 1997; Slatkin 1993], positive significant correlation between genetic and geographic distance found in our study is as expected.

### Comparative variation in host plant and associated invertebrates

*Urtica dioica* has been referred to as an invertebrate “super-host” as this plant provides food and shelter for large numbers of specialist and generalist insects, notably in the Lepidoptera, Coleoptera and Hemiptera [Davis 1989] as well as maintaining large populations of molluscs. However, surprisingly few genetic studies are available to allow a comparison of patterns of genetic diversity across host plant and associated invertebrates in this system. One study, using the small tortoiseshell butterfly (*Aglais urticae* L.), found high levels of gene flow and little or no population or geographic structure across the Palaearctic [Vandewoestijne et al. 2004], which the authors attributed to rapid population expansion facilitated by the ubiquitous and widespread distribution of the host. The relatively typical overall genetic divergence in *Trioza urticae* compared to other psyllid systems [Percy 2003, Taylor et al. 2016] suggests that range expansions are also facilitated by the abundant frequency and breadth of host distribution, but these are apparently more limited and less rapid than in the small tortoiseshell butterfly. Although the host plants were also sampled during collection of the nettle psyllids for this study, the observed morphological and cytological diversity in the nettle samples is complex [Cronk et al. 2016] and there are no obvious or clear associative patterns with the structure of variation in the nettle psyllid.

## Conclusions

Despite distinct regional structure in the genetic variation of *Trioza urticae*, overall genetic divergence, particularly considering the extremely large geographic range, is relatively small (<3%) and is well within intraspecific divergence reported for other psyllid taxa [Percy 2003, Taylor et al. 2016]. We therefore consider the observed variation in *Trioza urticae* to be typical and not suggestive of reproductive isolation indicative of incipient species divergence. However, local adaptation may be contributing to the maintenance of clinal variation and further study of the morphotypic variation associated with genetic variation and local environmental variables will be the subject of a following study.

## Figures and Tables

**Table 1.** Summary of sites sampled. Further information about sites can be found in Cronk et al. (2015).

Site no.	Country	Longitude	Latitude	No. of specimens sequenced
2	Greece	22.3102	38.9020	5
5	Greece	23.2739	41.1133	5
8	Bulgaria	23.8106	42.9240	5
11	Romania	23.1903	44.9620	5
14	Hungary	21.3127	46.7007	5
17	Poland	21.6975	49.4635	5
20	Poland	21.1971	51.7750	5
22	Poland	22.3030	53.5548	5
24	Lithuania	23.7742	54.9258	5
26	Latvia	24.2516	56.7114	5
29	Estonia	24.9358	59.4029	5
30	Finland	24.6584	60.2730	5
32	Finland	26.1237	62.0496	5
34	Finland	25.5266	64.0507	5
35	Finland	25.5381	64.6129	1
37	Finland	23.8945	66.2495	5
39	Finland	23.6341	67.9118	5
42	Norway	23.6658	70.6523	4
D-iGR	Greece	20.5231	39.2358	2
K-iiNL	Netherlands	6.0353	53.1508	5
L-FR	France	1.8551	50.8652	2
Eng	England, UK	0.2301	51.4386	2
